# Family resilience, emotional intelligence, and non-suicidal self-injury among Chinese adolescents with mental disorders: a latent variable mediation analysis

**DOI:** 10.3389/fpsyt.2025.1631266

**Published:** 2025-10-16

**Authors:** Zhengmin Zhu, Bican Tan, Yingqiong Ge, Chuan Li, Shuting Zou, Xiaojian Jiang

**Affiliations:** ^1^ College of Nursing, Hunan University of Chinese Medicine, Changsha, Hunan, China; ^2^ Child and Adolescent Psychology Department, Hunan Provincial Brain Hospital, Changsha, Hunan, China

**Keywords:** non-suicidal self-injury, family resilience, emotional intelligence, adolescent mental disorders, latent variable

## Abstract

**Background:**

Non-suicidal self-injury (NSSI) is a common malpractice in adolescents with mental disorders. It may lead to suicide or other adverse consequences, thus affecting the treatment and rehabilitation of patients. We herein analyzed the relationship among family resilience, emotional intelligence, and NSSI behavior in adolescents with mental disorders.

**Methods:**

We conducted a cross-sectional survey of 294 adolescent patients with mental disorders (91 boys and 203 girls) from the counselling center and inpatient adolescents of the Pediatric Psychology Department of Hunan Brain Hospital. Data were collected using the Family Resilience Scale, Emotional Intelligence Scale, and Adolescent Non-Suicidal Self-Injury Assessment Questionnaire. Structural equation modeling (SEM) was used to explore the mediating role of emotional intelligence in the association between family resilience and NSSI in these adolescents.

**Results:**

Herein, 229/294 patients reported at least one episode of NSSI behavior in the last one year. They had low levels of family resilience and emotional intelligence. Family resilience and emotional intelligence were significantly negatively correlated with NSSI behavior; consequently, they showed a significant association with NSSI behavior. The SEM analysis showed that emotional intelligence plays a partial mediating role in the relationship between family resilience and NSSI.

**Conclusion:**

Family resilience and emotional intelligence are important protective factors for NSSI behaviors in adolescent patients with mental disorders. Future research can focus on stimulating the ability of adolescent patients with mental disorders to combine the strengths of their own and their family’s resources, find the right direction for their individual development, and promote their treatment and recovery.

## 1 Introduction

With the gradually increasing incidence rates of psycho-behavioral problems and mental disorders in adolescents, mental disorders have become a major cause of illness, disability, and death in adolescents (1, 2). Non-suicidal self-injury (NSSI) refers to a series of repeated, intentional, and direct injuries to one’s own body without suicidal ideation; it has societal stigma associated with it, and the already high and still increasing incidence rates of NSSI in adolescents have aroused widespread concern in all sectors of the society (3). NSSI is reportedly closely associated with suicide (3) and is a significant predictor for the development of suicidal intent, suicidal behaviors, and long-term psychological disorders in adolescents in the future, and it is an important risk factor for predicting suicidality (4, 5). NSSI widely exists in patients with mental disorders, and the presence of a mental disorder makes the patient vulnerable to other mental disorders. Notably, mental disorders and NSSI interact with each other, which not only severely affects the physical and mental health of the individual but also causes significant disease burden to the family and society (6, 7). Studies have found the incidence of NSSI in adolescents with mental disorders to be very high at 37%–71% (8, 9). As of 2024, the current situation of NSSI in adolescents with mental disorders is not optimistic and needs to be urgently resolved.

During the exploration of the factors influencing NSSI in adolescents with mental disorders, family resilience and emotional intelligence have gained the attention of researchers. Family resilience refers to the process of family-based coping and adaptive functioning when a family member, an adolescent in the context of this study, is experiencing a traumatic or negative event (10). Family cohesion and family resilience scores were found to be significantly lower in adolescents with depression who had NSSI behavior than in those who did not have NSSI behavior; furthermore, in adolescent patients with mental disorders, family cohesion was significantly negatively correlated with NSSI, and there was a direct and significant correlation between family problems and NSSI involvement (11, 12). Emotional intelligence refers to an individual’s ability to perform accurate reasoning about emotions and the ability to utilize emotions and emotional knowledge to develop a better thought process (13). Self-injurious behavior is negatively correlated with emotional intelligence, and individuals with higher emotional intelligence are reportedly aware of their mental state and the consequences of their actions in time; they follow doctors’ instructions closely and cooperate with them better in case of an illness (14). At the same time, emotional intelligence is an important process between family risk and adolescent adjustment and can be impaired when family financial capacity and family conflict affect the development of family dynamics (15). Although the correlation of family resilience and emotional intelligence with NSSI has been studied, few studies have explored the mediators and underlying mechanisms of the relationship of family resilience and emotional intelligence with NSSI. In addition, few studies on family resilience, emotional intelligence, and NSSI have been conducted specifically for Chinese adolescents with mental disorders, whose perceptions of family resilience and emotional intelligence and their degree of development may be limited by the inter-disease maladaptive behavioral differences and socio-familial cultural influences.

Family systems theory suggests that mental problems faced by an individual could have risen from the dysfunctionality of interactions within the family environment. This theory emphasizes two variables (self-differentiation and chronic anxiety) and one concept (the triangle) and is centered on the causal loop of family interactions, wherein the perceptions and behaviors of family members are causally related to their interactions with each other (16, 17). It is important to note that children are less self-differentiated than their parents and have higher anxiety levels. Accordingly, in the process of the transmission of anxiety within the family, when too much anxiety is focused on one child, the child cannot regulate his or her own emotions and feelings, consequently leading to problematic behaviors. At the same time, a working family system has the ability to self-regulate, and crisis is not only a challenge but also an opportunity for such a family system (17, 18). According to a previously reported cognitive–emotional model, individuals experiencing emotional volatility develop five main characteristics: the tendency to react emotionally, negative self-schema, outcome expectations, insufficient confidence in self-injury and resistance to self-injury, and poor emotion regulation strategies. These collectively significantly increase the chances of the individual exhibiting NSSI behaviors to self-regulate emotional responses without external stimuli or undesirable situational risks (19). This model highlights that individuals may have stable emotional responses and/or attentional biases that influence how they perceive and interact with society (19). In addition, empirical research suggests that the relationship between emotional reactivity and NSSI is moderated by specific cognitions about self-injury, and people who believe they can resist NSSI behaviors and use other strategies to manage their emotions are less likely to engage in NSSI (20, 21). It suggests that believing in their ability to resist NSSI may be the key to preventing the onset and relapse of NSSI in adolescents.

The present study uses the family systems theory and cognitive–emotional model as a theoretical framework to explore the influence of family on individual behavior when adolescents are confronted with the uncomfortable stimuli associated with a mental disorder in the context of emotional activities, such as emotional triggers and stressors, contributing to the emergence of NSSI behaviors in the individual. Therefore, in this study, while taking into account the existing theoretical models and literature research, we performed a cross-sectional investigation to deeply analyze the mechanism of NSSI behaviors in terms of its association with family resilience and emotional intelligence in adolescents with mental disorders. We hope our findings open up new avenues to (i) reduce NSSI behaviors in adolescents with mental disorders, (ii) promote the physical and mental development and rehabilitation of adolescents with mental disorders, and (iii) provide theoretical support and decision-making basis for policymakers when drafting policies on the prevention, management, and rehabilitation of NSSI behaviors and provisions of assistance to adolescents with mental disorders exhibiting NSSI behaviors.

## 2 Materials and methods

### 2.1 Participants

In this study, we enrolled adolescent patients with mental disorders who visited the Pediatric Psychiatry Inpatient and Counselling Centre of the Pediatric Psychology Department of Hunan Brain Hospital between May 2023 to July 2023 and completed a questionnaire anonymously.

The following were the inclusion criteria: (1) age of 12–18 years (contains critical value); (2) ≥6 years of education; (3) diagnosis established as per the “International Classification of Diseases (11th Edition) (Code 6A20: Schizophrenia; 6A60-6A61: Bipolar I Disorder and Bipolar II Disorder; 6A70-6A71: Single Episode Depressive Disorder and Recurrent Depressive Disorder)” (ICD-11) criteria for mental disorders; (4) mental illness symptoms assessed using the Brief Psychiatric Rating Scale, with the score being <35 points; and (5) written informed consent obtained from patient and family members before participating in the project.

The exclusion criteria were the presence of (1) severe physical disease, (2) severe neurological disorders or poor cognitive function making it difficult to complete the questionnaire, and (3) self-reported suicidal intent or researchers perceiving the patient’s self-injurious behavior as life-threatening.

### 2.2 Procedure

Before data collection, the hospital and its various departments were informed about the study, their unanimous consent was obtained, the questionnaire was developed on the basis of a review of relevant literature and previous studies, the scientific nature of the questionnaire and minimum burden on the included patients were ensured, and a survey team was formed. We communicated effectively with patients during the data collection process to build a rapport and make them feel comfortable with the study. The assessment time was 20~30 min; if the patients struggled with understanding any part of the questionnaire, their queries were promptly clarified, and the questionnaires were collected and checked immediately after completion. The study was approved by the Ethics Committee of Hunan Brain Hospital (Approval No. 2021K056) and was performed in compliance with the Declaration of Helsinki. Adolescent patients with mental disorders or their guardians provided a signed informed consent form before participation in the study.

### 2.3 Measures

#### 2.3.1 General demographic data

The parameters for which data were to be collected were finalized by the researchers by referring to previous research performing a literature review. These parameters included age, gender, primary family caregiver, parental marital status, siblings, and left-behind experiences (Left-behind experiences refer to a situation where a child is raised by their grandparents in economically disadvantaged areas while the parents pursue employment opportunities in more developed regions. This skip-generation parenting creates a psychologically disadvantageous situation as these children do not live with their parents.).

#### 2.3.2 Family resilience chart

Qian Yongchun developed this on the basis of the Family Skills Inventory (FSI) by Orthner et al. (22, 23). This contains 33 questions in total, with each question scored on a four-point scale. Accordingly, the total possible score ranges from 33 to 132, with higher scores indicating higher levels of trauma exposure. The scale was reverse scored to make the results of the study more reliable. In addition, the values were calculated to be positively scored to facilitate the statistical analysis of the study, with higher scores indicating better family resilience. The questionnaire was completed by the patients or their guardians and interpreted by the researcher as necessary. The Cronbach’s α for the total FRC score in this study was 0.912 and the KMO was 0.904.

#### 2.3.3 Emotional intelligence scale

This scale was based on Salovey and Mayer’s (1990) model of emotional intelligence, which was a self-report questionnaire developed by Schutle et al. (24). The emotional intelligence scale by Schutle et al. (24). comprises 33 items across 4 dimensions, namely emotional understanding, emotional perception, emotional regulation, and emotional use. The Chinese version was translated and revised by Wang and shows good reliability and validity in adolescents (25). The questions were scored on a scale of 1–5, with 1 representing *strongly disagree* to 5 representing *strongly agree*; higher scores indicated higher levels of emotional intelligence in individuals. With a Cronbach’s α of 0.89 in this study, the EIS scale showed good reliability and validity.

#### 2.3.4 Adolescent non-suicidal self-injury assessment questionnaire

This questionnaire was developed by Wan et al. and covers two dimensions, namely behavioral and functional questionnaires, with 12 and 19 items in the former and the latter, respectively (26). The questions were scored on a five‐point Likert scale, with 1 representing *never* and 5 representing *always*. Higher scores indicated more severe self‐injury. In this study, we mainly used responses to the behavioral questionnaire for our analyses. With a Cronbach’s α of 0.93 in this study, the behavioral questionnaire showed good reliability and validity.

### 2.4 Analytic procedures

Preliminary data analyses were conducted using SPSS 25.0^®^ (IBM, Armonk, NY, USA). All variables were entered into Excel by two authors and processed and statistically analyzed using SPSS. All variables showed complete data, thanks to strict data collection measures. Normally distributed data were expressed as $\bar{\text{x}}$ ± *s*. For comparison of differences between groups when the groups show normal distribution, the chi-square test and independent samples t-test were used. One-way analysis of variance (ANOVA) was used for multi-group comparisons. When continuous variables were non-normally distributed, M(QR) was used to describe the statistic, and non-parametric test was used for comparing between-group differences. The Mann–Whitney U test was used for comparing two independent samples, and the Kruskal–Wallis H rank sum test was used for comparing multiple independent samples. The Dunn’s method was used for two-by-two comparisons when between-group differences were identified. Correlation analyses between scores were performed using the Spearman’s correlation coefficient. Latent variable structural equation modelling (SEM) was used to analyze the mediating effect of emotional intelligence on the relationship between family resilience and NSSI behaviors. The Hayes PROCESS macro (Model 4) was used to test the mediating effect, with 5,000 bootstrap samples and the calculation of bias-corrected 95% confidence intervals (27). All modelling procedures were performed using IBM^®^ SPSS^®^ Amos™ 22 (IBM). The following indices determined the goodness of fit of the model: Chi-square ratio of degrees of freedom (χ^2^/df) < 2 and *P* > 0.05, the root mean square error of approximation (RMSEA) < 0.08, the Comparative Fit Index (CFI) > 0.90, the adjusted goodness-of-fit index (AGFI) > 0.90, the normed fit index (NFI) > 0.90, and the Tucker–Lewis index (TLI) > 0.90. A two-tailed *P* value of < 0.05 indicated significant difference.

## 3 Results

### 3.1 Descriptive statistics

Sociodemographic characteristics of participants are shown in **Table 1**; the study cohort comprised 91 males (31%) and 203 females (69%) aged 12–18 years (mean ± SD = 14.54 ± 1.51 years). Overall, for 79.3% of the participants, their parents were in their first marriage, and for 49.3% of the participants, their primary caregiver was their mother. Most patients had siblings (78.2%), and 78.9% of patients had no left-behind experience.

**Table 1:** Sociodemographic characteristics of participants (*N* = 294).

Variables	Categories	N (%)	$\bar{\text{x}}$ ± *s/M* (*Q_R_*)	Significant effect *P* < 0.05
Age			14.54 ± 1.51	E, N
Gender	Male	91 (31)		F, E, N
	Female	203 (69)		
Parental Marital status	Married	233 (79.3)		F, N
	Remarriage	18 (6.1)		
	Divorced	31 (10.5)		
	Others	12 (4.1)		
Primary caregivers	Parents	62 (21.1)		F, E, N
	Father	37 (12.6)		
	Mother	145 (49.3)		
	Grandparents	37 (12.6)		
	Others	13 (4.4)		
Siblings	Yes	230 (78.2)		
	No	64 (21.8)		
Left-behind experiences	Yes	62 (21.1)		F, E
	No	232 (78.9)		
FSC			56.09 ± 10.75	
EIS			112.46± 17.33	
NSSI			9 (16.25)	

Independent t-test and ANOVA were used to calculate the differences of FSC and EIS in various demographic variables; the Mann–Whitney U test and Kruskal–Wallis H rank sum test were used to calculate the differences in NSSI behaviors for various demographic variables (F = FSC; E = EIS; N = NSSI). FSC, Family Resilience Chart; EIS, Emotional Intelligence Scale; NSSI, non-suicidal self-injury.

The mean total FSC and EIS scores and NSSI behaviors were (56.09 ± 10.75), (112.46 ± 17.33), and 9 (16.25), respectively. Furthermore, there were significant differences between FSC and EIS scores and NSSI behaviors with respect to the following general statistics: age, gender, parental marital status, primary caregivers, siblings, and left-behind experiences. In particular, significant differences in FSC and EIS scores and NSSI behaviors were noted between the two genders, with women exhibiting more NSSI behaviors and having lower FSC and EIS scores than men. FSC scores and NSSI behaviors significantly differed with the parental marital status, with patients having first-time married parents showing higher family resilience scores and lesser self-injurious behavior than patients having parents with other marital statuses (remarriage, divorced, separated, and widowed). Besides, FSC and EIS scores for NSSI behaviors significantly differed depending on who the primary caregiver was, and FSC and EIS significantly differed depending on the left-behind experiences of patients.

We also compared the psycho-social characteristics of the female and male adolescents (**Table 2**). There were statistically significant differences between male and female adolescents with mental disorders in terms of age group, parental marital status, primary caregiver, presence or absence of NSSI behavior, emotional intelligence scores, and family resilience scores (*P* < 0.05).

**Table 2:** Characteristics of male and female adolescents (*N* = 294).

Variables	Categories	Male (*N* = 91)	Female (*N* = 203)	*P*
Age	12–13	15	70	0.001*
	14–15	37	82	
	16–18	39	51	
Parental Marital status	Married	70	163	0.040
	Remarriage	6	12	
	Divorced	7	24	
	Others	8	4	
Primary caregivers	Parents	27	35	0.011*
	Father	17	20	
	Mother	33	112	
	Grandparents	10	27	
	Others	4	9	
Siblings	Yes	71	109	0.500
	No	20	44	
Left-behind experiences	Yes	17	45	0.498
	No	74	158	
NSSI	Yes	55	174	<0.001*
	No	36	29	
FSC		58.74 ± 10.70	54.90 ± 10.58	0.004*
EIS		117.56 ± 16.69	110.18 ± 17.16	<0.001*

*Statistically significant at *P* < 0.05 level, two-sided.

### 3.2 Correlation analysis

We identified a significant negative correlation between NSSI behaviors and FSC and EIS scores (*r_FRC_* = -0.425, *P* < 0.01 and *r_EIS_* = -0.418, *P* < 0.01, respectively). Family resilience was found to be significantly and positively related to emotional intelligence (*r* = 0.379, *P* < 0.01; **Table 3**).

**Table 3:** Correlations of the variables of FSC and EIS scores with NSSI behaviors.

Variables	$\bar{\text{x}}$ ± *s/M* (*P_25_*, *P_75_*)	FRC	F1	F2	F3	F4	F5	F6	EIS	E1	E2	E3	E4	NSSI	N1	N2
FRC	56.09 ± 10.75	1														
F1	13.56 ± 2.14	0.342^**^	1													
F2	7.74 ± 2.30	0.843^**^	0.223^**^	1												
F3	8.17 ± 2.27	0.857^**^	0.159^**^	0.749^**^	1											
F4	9.86 ± 3.34	0.860^**^	0.048	0.707^**^	0.717^**^	**1**										
F5	7.78 ± 2.16	0.733^**^	0.086	0.533^**^	0.572^**^	0.608^**^	1									
F6	8.97 ± 2.02	0.782^**^	0.289^**^	0.594^**^	0.642^**^	0.584^**^	0.580^**^	1								
EIS	112.46 ± 17.33	0.379^**^	0.101	0.336^**^	0.345^**^	0.394^**^	0.189^**^	0.321^**^	1							
E1	40.30 ± 7.06	0.267^**^	0.014	0.250^**^	0.274^**^	0.270^**^	0.158^**^	0.197^**^	0.817^**^	1						
E2	26.68 ± 5.15	0.406^**^	0.087	0.355^**^	0.367^**^	0.422^**^	0.216^**^	0.351^**^	0.828^**^	0.514^**^	1					
E3	20.91 ± 3.87	0.211^**^	0.071	0.156^**^	0.180^**^	0.221^**^	0.112	0.207^**^	0.721^**^	0.492^**^	0.504^**^	1				
E4	24.57 ± 5.03	0.291^**^	0.145^*^	0.252^**^	0.227^**^	0.297^**^	0.114	0.273^**^	0.832^**^	0.503^**^	0.725^**^	0.544^**^	1			
NSSI	9 (2,18.25)	-0.425^**^	-0.107	-0.333^**^	-0.344^**^	-0.447^**^	-0.320^**^	-0.372^**^	-0.418^**^	-0.255^**^	-0.454^**^	-0.199^**^	-0.403^**^	1		
N1	7 (1,12)	-0.412^**^	-0.104	-0.315^**^	-0.343^**^	-0.428^**^	-0.327^**^	-0.361^**^	-0.397^**^	-0.231^**^	-0.427^**^	-0.204^**^	-0.391^**^	0.973^**^	1	
N2	2 (0,6)	-0.371^**^	-0.095	-0.304^**^	-0.283^**^	-0.397^**^	-0.254^**^	-0.328^**^	-0.411^**^	-0.264^**^	-0.450^**^	-0.169^**^	-0.389^**^	0.883^**^	0.766^**^	1

**P < 0.01, and *P* < 0.05 are marked in boldface.

FRC, Family Resilience Chart; F1, family economics; F2, family cohesion; F3, family beliefs; F4, family communication; F5, social support for family; F6, Family’s problem-solving ability; EIS, Emotional Intelligence Scale; E1, emotion perception; E2, management of self-emotion; E3, emotional management of others; E4, utilization of emotions; NSSI, non-suicidal self-injury; N1, non-suicidal self-injurious behavior of non-obvious tissue injury; N2, non-suicidal self-injurious behavior of obvious tissue injury.

### 3.3 Regression analysis

On various dimensions of FSC, social support for family had a significant impact on NSSI. On various dimensions of EIS, management of self-emotion, emotional management of others, utilization of emotion had a significant impact on NSSI. Among the covariates, gender, parental marital status (married vs. others), primary caregivers (mother vs. father), According to the diagnostic analysis of covariance, there was no significant multicollinearity among family resilience, emotional intelligence, NSSI behavior, gender, parental marital status, and primary caregiver variables. The results are shown in **Table 4**.

**Table 4:** Linear regression analysis of FSC and EIS scores with NSSI behaviors.

	Predictor variables	Group	Reference	*B*	*SE*	*β*	v*t*	*P*	VIF
Independent variables	Constant			46.672	7.547		6.185	<0.001	
	FRC								
	F1			-0.322	0.267	-0.061	-1.203	0.230	1.186
	F2			0.093	0.395	0.019	0.235	0.815	2.972
	F3			-0.039	0.424	-0.008	-0.091	0.928	3.342
	F4			-0.308	0.250	-0.091	-1.233	0.219	2.519
	**F5**			**-0.678**	**0.335**	**-0.129**	**-2.023**	**0.044**	**1.893**
	F6			-0.163	0.400	-0.029	-0.408	0.683	2.366
	EIS								
	E1			-0.064	0.096	-0.040	-0.662	0.509	1.662
	**E2**			**-0.548**	**0.168**	**-0.249**	**-3.255**	**<0.001**	**2.708**
	**E3**			**0.392**	**0.178**	**0.134**	**2.208**	**0.028**	**1.710**
	**E4**			**-0.373**	**0.169**	**-0.166**	**-2.206**	**0.028**	**2.618**
Concomitant variable	Age			-0.729	0.371	-0.097	-1.966	0.050	1.130
	**Gender**			**4.166**	**1.236**	**0.170**	**3.371**	**<0.001**	**1.182**
	Parental Marital status	Remarriage	Married	-3.775	2.390	-0.080	-1.579	0.115	1.189
		Divorced		0.661	1.891	0.018	0.349	0.727	1.222
		**Others**		**-5.774**	**2.834**	**-0.101**	**-2.037**	**0.043**	**1.139**
	Primary caregivers	Parents	Mother	-2.125	1.434	-0.077	-1.482	0.140	1.240
		**Father**		**6.316**	**1.800**	**0.185**	**3.509**	**0.001**	**1.291**
		Grandparents		3.360	1.753	0.099	1.916	0.056	1.225
		Others		0.146	2.666	0.003	0.055	0.956	1.088
	R^2^	0.407							
	F	9.915							
	P	< 0.001							
Dependent variables	NSSI								

*P < 0.05, **P < 0.01, and *P* < 0.05 are marked in boldface.

VIF, Variance inflation factor (VIF < 10, there is no serious multicollinearity in variables). Gender (male = “1”, female = “2”). FRC, Family Resilience Chart; F1: family economics; F2, family cohesion; F3, family beliefs; F4, family communication; F5, social support for family; F6, family’s problem-solving ability; EIS, Emotional Intelligence Scale, E1, emotion perception; E2, management of self-emotion; E3, emotional management of others; E4, utilization of emotion; NSSI, non-suicidal self-injury.

### 3.4 Mediation analysis

**Figure 1:**
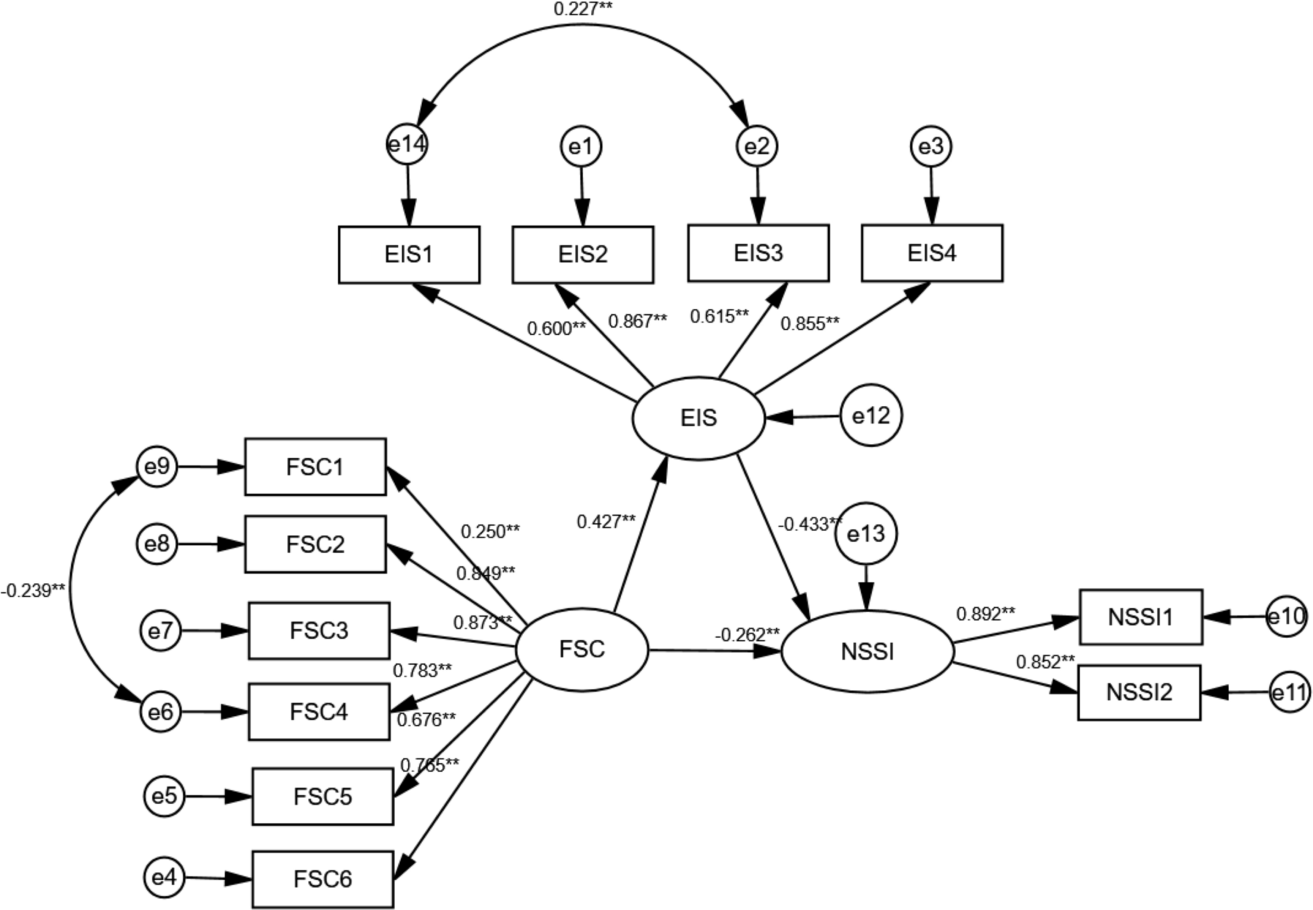
Mediation effect of emotional intelligence on the link between family resilience and NSSI (**P < 0.01).

Latent variable SEM was used to test the mediating role of emotional intelligence in the relationship between family resilience and NSSI (**Figure 1**). The hypothetical model fit the data well: χ^2^ */df* = 2.337 (*P* < 0.01), RMSEA = 0.068 (95% *CI*: 0.051, 0.084), CFI = 0.963, AGFI = 0.907, NFI = 0.938, and TLI = 0.950). The standardized path coefficients from family resilience to emotional intelligence (*β* = 0.427, *P* < 0.001) and from emotional intelligence to NSSI (*β* = -0.433, *P* < 0.001) were statistically significant. Moreover, the direct effect of family resilience on NSSI was significant (*β* = -0.262, *P* < 0.001). The total effect in this model was -0.447, and the mediating effect was -0.185, which accounted for 41.4% of the total effect. Adolescents with mental disorders who have lower family resilience experience lower emotional intelligence, which may increase NSSI behaviors (**Table 5**).

**Table 5:** The path analysis results of emotional intelligence between family resilience and non-suicidal self-injury.

Project		Standardized effect value	SE	95% CI	Relative effect value
Direct effect	Family resilience → NSSI behavior	-0.262	0.077	(-0.407, -0.106)	58.6%
Indirect effect	Family resilience → Emotional intelligence → NSSI behavior	-0.185	0.048	(-0.295, -0.106)	41.4%
Total effect		-0.447	0.058	(-0.553, -0.324)	

## 4 Discussion

NSSI is a common and serious mental health problem in adolescents with mental disorders. With increasing research focus on positive psychology, studies on protective factors of NSSI behaviors in adolescents with mental disorders have been increasing. Early studies explored the influence of family resilience and emotional intelligence on NSSI behaviors in adolescents with mental disorders; however, the mechanism underlying this influence is under-researched, and thus, in the present study, we used structural equation modeling to investigate how family resilience and emotional intelligence affect the NSSI of adolescents with mental disorders. The results showed that family resilience affects NSSI behaviors in adolescents with mental disorders directly as well as indirectly through emotional intelligence. These results provide a basis for further exploration of the prevention and rehabilitation of NSSI in adolescents with mental disorders.

In this study sample, the proportion of females was higher than that of males. This may be attributed to two factors, with the first being gender differences inherent in the study design. The study primarily included patients with mood disorders, and previous research has shown through genomic analysis that women have a higher risk of developing mood disorders, whereas men have a higher risk of developing behavioral disorders (28). The second factor is culturally related gender differences. Due to deeply ingrained traditional social roles and academic pressure, female adolescents often face constraints like educational inequality and low expectations (29). In addition, this study revealed statistically significant gender differences in the primary caregivers of adolescent mental disorder patients. Females who experienced paternal absence during early childhood exhibited higher levels of depressive symptoms throughout adolescence than their peers who had a father present (30). This study shows that the self-harm rate in adolescents with mental disorders is as high as 77.9%, with a significantly greater proportion of female patients showing self-harm behaviors (85.7%) than male patients (60.4%). This disparity may be attributed to biological and social environmental factors (27–32). First, it may be related to gender differences in hormones and emotional expression; second, it may be influenced by social and cultural environments. Asian cultures often view modesty and self-effacement as cultural virtues, suppressing the expression of positive emotions. In addition, gender role socialization in China leads to differences in how NSSI is expressed across genders. Women are expected to be gentle and reserved, whereas men are expected to be strong and independent. As a result, women often engage in internalized depressive self-harm behaviors (e.g., scratching, biting, or cutting), whereas men tend to use externalized impulsive self-harm behaviors (e.g., head-banging against hard objects). Furthermore, due to the unique nature of mental illnesses, women have a higher rate of mental health service utilization.

In the present study, we identified that adolescents with mental disorders have lower levels of family resilience and emotional intelligence. Upon exploring the reasons for this, we believe this variability may be attributed to our participants being adolescents with mental disorders, which can disturb the normalcy in family life and damage relationships. Furthermore, for adolescents, their parents are the linchpins of the family system, and as the mental health of the parents and their child developmentally interacts with each other, mental health interventions should also target the entire family system and not just the child (33). Moreover, the study participants were adolescents with a mental illness and inadequate emotional regulation, making them more susceptible to the emotions of others (34). In the present study, we found that the incidence of NSSI behaviors in adolescents with mental disorders was high, which is consistent with the findings of Lenkiewicz et al. (35). The higher incidence rates of NSSI behaviors in this study than in other previous studies in China (36) may be attributed to the following. First, the adolescents included in this study were from a tertiary psychiatric hospital in Hunan Province. These patients were admitted to the hospital and generally had high mood fluctuations, with some of them admitted to the hospital for recurrent self-injurious behaviors. Second, it may be attributed to the way the NSSI behavior was measured and number of participants enrolled.

Using regression analysis, this study found that emotional management of others (E3) was positively correlated with self-harming behavior (NSSI), whereas other dimensions of emotional intelligence were negatively correlated with it. This phenomenon may be partly influenced by sociocultural factors. Adolescents in their teenage years are in a phase of emotional socialization. The “other-oriented” perspective in traditional East Asian culture emphasizes the perception of others’ emotions, tending towards compliance with and consideration for others (37). Related studies indicate that Chinese adolescents score significantly higher on the dimension of attention to others’ emotions than on the dimension of self-emotional analysis, with weaker emotional self-awareness compared to sensitivity to others’ emotions. This suggests that adolescents are more skilled at capturing external emotional cues but still need to develop their emotional self-awareness (38). Unlike the EIS dimensions of emotional perception and self-regulation, managing others’ emotions (E3) requires externalized emotional labor. Therefore, increased responsibility of emotional management of others may lead to weakened self-emotional management in adolescents with mental disorders, promoting further development of NSSI behavior and thus posing a particular challenge to this population.

In this study, the findings of correlation analysis concluded that family resilience of adolescent mental disorders was significantly negatively correlated with NSSI behaviors. This was particularly true for family communication and family’s problem-solving ability dimensions but not for the family economics dimension, which was not found to be correlated with NSSI, probably because of China’s economic development and the implementation of poverty alleviation policies. Our findings of family resilience being significantly negatively correlated with NSSI behaviors in adolescents with mental disorders are corroborated by previous reports (39). We believe that these findings can be attributed to the protective force of family resilience emanating from the interaction between the individual and the family. When the family as a system has better economic capacity, family members can more effectively communicate with each other, making them a cohesive unit. This enables the family to more strongly believe in and support the adolescent in their family, thus increasing the likelihood of them finding and solving the problem and consequently reducing NSSI behaviors in the adolescent. In adolescent patients with mental disorders, emotional intelligence was significantly negatively correlated with NSSI behaviors, particularly for the management of self-emotions. First, the reason for this is related to altered neurobiological mechanisms. The current neuroimaging manifestations of NSSI are mainly in the amygdala and cingulate and prefrontal cortex in the limbic system, which are involved in emotion perception processing and emotion regulation (40). Studies have shown enhanced activation of the amygdala, insula, and anterior cingulate cortex in borderline personality disorder patients with NSSI behaviors, and enhanced amygdala activity is positively correlated with emotion regulation deficits (41). In addition, in a study assessing differences in emotional processing between adolescents with and without NSSI, it was found that the NSSI group showed increased activation of the amygdala and anterior cingulate gyrus in response to emotional pictures and showed significantly stronger brain responses (42). Second, the cognitive–emotional model states that emotional responses and processing influence how an individual responds to and processes stimuli, and NSSI is a single outcome of “poor” emotion regulation (19). Positive correlations between family resilience and emotional intelligence in adolescent patients with mental disorders uphold the notion that family is crucial to adolescent emotional development; a positive family climate is associated with neural activation of adolescent emotional responses and regulation (43). Patients with highly resilient families can better control their emotions, thus helping them correctly adjust to negative emotions and positively process negative events (44). Therefore, family resilience and emotional intelligence are important protective factors for NSSI behaviors in adolescents with mental disorders, with a strong buffering effect.

In this study, the mediating effect of emotional intelligence on the relationship between family resilience and NSSI behaviors was analyzed using structural equation modelling, which showed that emotional intelligence partially mediated the relationship between family resilience and NSSI, which is consistent with the findings of Boyes et al. (45). For individuals with low family resilience, lower emotional intelligence scores were associated with a higher likelihood of self-injurious behavior. When an adolescent in a family develops a mental illness, a series of conflicts may arise in the family system, and family members are likely to experience a series of negative emotions and feelings of self-blame; this can consequently strengthen the caregiver’s control over the adolescent, which the adolescent is likely to perceive as “invasive” behavior. This coupled with the fact that adolescents with mental disorders are subject to the influence of the illness and cognitive changes limits their ability to regulate emotions. Under these circumstances, they may experience uncontrollable emotions and consequently resort to NSSI behaviors to alleviate their negative emotions.

### 4.1 Strengths and limitations

Our research focuses on protective factors for NSSI, examining the family as a whole to gain a deeper understanding of how social environmental factors and individual emotional factors influence NSSI behavior in adolescents with mental disorders. The study aims to promote a close connection between family resilience and emotional intelligence in adolescents, providing targeted guidance on how individuals can think and act when facing adversity, thus helping them better adapt to adverse environments and enhance their ability to cope with challenges. Our study has several limitations. First, this study is a cross-sectional study. The analysis of the information derived from the research subject is quite subjective and does not allow the inference of a causal relationship between the variables. Furthermore, the study did not stratify the different types of mental disorders and had inadequate control of confounding factors. Second, although we identified that emotional intelligence has a mediating role in the relationship between family resilience and NSSI, emotional intelligence was a partial mediator, and the influence of other variables on the self-injurious behaviors of the patients’ needs to be further investigated. Third, during our in-depth literature search for the current study, we found that intentional and repeated ingestion of foreign objects by adolescent patients with mental disorders is also gradually becoming an NSSI behavior of concern. This modality of expression causes wastage of medical resources and increases the burden on families, medical care systems, and society; however, there is currently insufficient research-based knowledge about this modality.

## 5 Conclusion

Our research found that family resilience and emotional intelligence are important protective factors for NSSI. Adolescence is a critical stage in the development of an individual’s cognitive, emotional, and social skills. Adolescents are the key to national development and the future of the nation, and thus, the prevention and treatment of adolescent mental disorders are of great significance. This study found the prevalence of self-harming behaviors to be relatively high in adolescents with mental disorders, indicating that medical professionals and social workers should guide such patients in effective emotional management and enhance their emotional intelligence, while considering the differences in individual emotional development and family structures within China’s socio-cultural context. In addition, they should advise family members to focus on the overall development of the family, create a positive family environment, and shift the resilience strategies of adolescents with mental health issues from “inadequate and chaotic” to “effective and mature.” This study provides a reference for developing clinical intervention plans targeting self-harm behaviors in adolescents with mental health issues, as well as designing targeted and appropriate preventive and therapeutic measures. Future research can focus on (i) stimulating the ability of adolescents with mental disorders to integrate their own and their families’ strengths and resources, (ii) identifying the right direction for individual development and conducting targeted research on NSSI behavior in individuals with different types of mental illnesses, and (iii) providing references for the design of behavioral intervention programs to reduce NSSI in adolescents with mental disorders in the future. This will lead to more targeted and appropriate preventive and curative measures to guide the development of family resilience and good mood of adolescents with mental disorders, as well as to promote the sustainable development of adolescent mental and psychological health.

## Data Availability

The raw data supporting the conclusions of this article will be made available by the authors, without undue reservation.

## References

[1] Li F Cui Y Li Y Guo L Ke X Liu J. Prevalence of mental disorders in school children and adolescents in China: diagnostic data from detailed clinical assessments of 17,524 individuals. J Child Psychol Psychiatry. (2022) 63:34–46. doi: 10.1111/jcpp.13445, PMID: 34019305

[2] Mental Health. World mental health report: transforming mental health for all. Geneva: World Health Organization (2022).

[3] Nock MK Favazza AR. Nonsuicidal self-injury: definition and classification. Am psychol Assoc. (2009) 9–18. doi: 10.1037/11875-001

[4] Wang C Zhang P Zhang N. Adolescent mental health in China requires more attention. Lancet Public Health. (2020) 5:e637. doi: 10.1016/s2468-2667(20)30094-3, PMID: 33271076

[5] Baer MM LaCroix JM Browne JC Hassen HO Perera KU Weaver J. Non-suicidal self-injury elevates suicide risk among United States military personnel with lifetime attempted suicide. Arch Suicide Res. (2018) 22:453–64. doi: 10.1080/13811118.2017.1358225, PMID: 28885089

[6] Steenkamp LR de Neve-Enthoven NGM João AM Bouter DC Hillegers MHJ Hoogendijk WJG. Psychotic experiences, suicidality and non-suicidal self-injury in adolescents: Independent findings from two cohorts. Schizophr Res. (2023) 257:50–7. doi: 10.1016/j.schres.2023.05.006, PMID: 37285715

[7] Hielscher E Connell M Lawrence D Zubrick SR Hafekost J Scott JG. Association between psychotic experiences and non-accidental self-injury: results from a nationally representative survey of adolescents. Soc Psychiatry Psychiatr Epidemiol. (2019) 54:321–30. doi: 10.1007/s00127-018-1629-4, PMID: 30478528

[8] Liu RT Walsh RFL Sheehan AE Cheek SM Sanzari CM. Prevalence and correlates of suicide and nonsuicidal self-injury in children: A systematic review and meta-analysis. JAMA Psychiatry. (2022) 79:718–26. doi: 10.1001/jamapsychiatry.2022.1256, PMID: 35612875 PMC9134039

[9] Chen Y Fu W Ji S Zhang W Sun L Yang T. Relationship between borderline personality features, emotion regulation, and non-suicidal self-injury in depressed adolescents: a cross-sectional study. BMC Psychiatry. (2023) 23:293. doi: 10.1186/s12888-023-04800-1, PMID: 37118709 PMC10148398

[10] Walsh F. The concept of family resilience: crisis and challenge. Fam Process. (1996) 35:261–81. doi: 10.1111/j.1545-5300.1996.00261.x, PMID: 9111709

[11] Shao C Wang X Ma Q Zhao Y Yun X. Analysis of risk factors of non-suicidal self-harm behavior in adolescents with depression. Ann Palliat Med. (2021) 10:9607–13. doi: 10.21037/apm-21-1951, PMID: 34628886

[12] Gyori D Farkas BF Horvath LO Komaromy D Meszaros G Szentivanyi D. The association of nonsuicidal self-injury with quality of life and mental disorders in clinical adolescents-A network approach. Int J Environ Res Public Health. (2021) 18:1840. doi: 10.3390/ijerph18041840, PMID: 33672808 PMC7918829

[13] Mayer JD Roberts RD Barsade SG. Human abilities: emotional intelligence. Annu Rev Psychol. (2008) 59:507–36. doi: 10.1146/annurev.psych.59.103006.093646, PMID: 17937602

[14] Tsirigotis K Łuczak J. Manifestations of indirect self-destructiveness and dimensions of emotional intelligence. Psychiatr Q. (2016) 87:377–86. doi: 10.1007/s11126-015-9396-9, PMID: 26453559 PMC4945676

[15] Cheung RYM Leung MC Chung KKH Cheung HY. Family risks and adolescent adjustment in Chinese contexts: testing the mediating role of emotional intelligence. J Child Fam Stud. (2018) 27:3887–96. doi: 10.1007/s10826-018-1233-y

[16] Zhang ZX. The development and current situation of family system theory. psychol Exploration. (1990) 20:31–4.

[17] Bowen M. Family Therapy in Clinical Practice. New York: Jason Aronson (1978).

[18] Xu HM Sheng XC. Family Therapy-Theory and Practice. Beijing, China: People’s Health Publishing House (2010).

[19] Hasking P Whitlock J Voon D Rose A. A cognitive-emotional model of NSSI: using emotion regulation and cognitive processes to explain why people self-injure. Cognit Emot. (2017) 31:1543–56. doi: 10.1080/02699931.2016.1241219, PMID: 27702245

[20] Duncan-Plummer T Hasking P Tonta K Boyes M. Cognitive-emotional networks in students with and without a history of non-suicidal self-injury. J Affect Disord. (2023) 329:394–403. doi: 10.1016/j.jad.2023.02.054, PMID: 36828146

[21] Dawkins JC Hasking PA Boyes ME Greene D Passchier C. Applying a cognitive-emotional model to nonsuicidal self-injury. Stress Health. (2019) 35:39–48. doi: 10.1002/smi.2837, PMID: 30221443

[22] Qian YC. The research on case work in improving the family resilience of single mothers family. Fuzhou University. (2017) 22–43.

[23] Orthner DK Cole G. (1999). An index of family strength: Measurement and trends, in: the annual meeting of the National Council on Family Relations, Los Angeles, California.

[24] Schutte NS Malouff JM Hall LE Haggerty DJ Cooper JT Golden CJ. Development and validation of a measure of emotional intelligence. Pers Individ Dif. (1998) 25:167–77. doi: 10.1016/S0191-8869(98)00001-4

[25] Wang CK. Emotional intelligence, general self-efficacy and coping style of delinquent teenagers. Chin Ment Health J. (2002) 8:566–7. doi: 10.3321/j.issn:1000-6729.2002.08.022

[26] Wan YH Liu W Hao JH Tao FB. Development and evaluation on reliability and validity of adolescent non-suicidal self-injury assessment questionnaire. Chin J School Health. (2018) 39:170–3. doi: 10.16835/j.cnki.1000-9817.2018.02.005

[27] Hayes AF. Partial, conditional, and moderated moderated mediation: Quantification, inference, and interpretation. Communication Monogr. (2018) 85:4–40. doi: 10.1080/03637751.2017.1352100

[28] Dong W Liu Y Bai R Zhang L Zhou M. The prevalence and associated disability burden of mental disorders in children and adolescents in China: a systematic analysis of data from the Global Burden of Disease Study. Lancet Reg Health West Pac. (2025) 55:101486. doi: 10.1016/j.lanwpc.2025.101486, PMID: 39995764 PMC11849651

[29] Ge X Conger RD Elder GH Jr. Pubertal transition, stressful life events, and the emergence of gender differences in adolescent depressive symptoms. Dev Psychol. (2001) 37:404–17. doi: 10.1037//0012-1649.37.3.404, PMID: 11370915

[30] Culpin I Heuvelman H Rai D Pearson RM Joinson C Heron J. Father absence and trajectories of offspring mental health across adolescence and young adulthood: Findings from a UK-birth cohort. J Affect Disord. (2022) 314:150–9. doi: 10.1016/j.jad.2022.07.016, PMID: 35842065 PMC10666570

[31] Law S Liu P. Suicide in China: unique demographic patterns and relationship to depressive disorder. Curr Psychiatry Rep. (2008) 10:80–6. doi: 10.1007/s11920-008-0014-5, PMID: 18269899

[32] Zhang SC Tao FB Wu XY Tao SM Fang J. Low health literacy and psychological symptoms potentially increase the risks of non-suicidal self-injury in Chinese middle school students. BMC Psychiatry. (2016) 16:327. doi: 10.1186/s12888-016-1035-y, PMID: 27650034 PMC5028961

[33] Speyer LG Hall HA Hang Y Hughes C Murray AL. Within-family relations of mental health problems across childhood and adolescence. J Child Psychol Psychiatry. (2022) 63:1288–96. doi: 10.1111/jcpp.13572, PMID: 35075634 PMC9787478

[34] Qi J Yu L Wang SM Wang ZZ. Disorder and reconstruction in daily life of adolescents with mental disorders. Med Philosophy. (2022) 43:52–6. doi: 10.12014/j.issn.1002-0772.2022.06.10

[35] Lenkiewicz K Racicka E Bryńska A. Self-injury - placement in mental disorders classifications, risk factors and primary mechanisms. Review of the literature. Psychiatr Pol. (2017) 51:323–34. doi: 10.12740/pp/62655, PMID: 28581540

[36] Xie X Li Y Liu J Zhang L Sun T Zhang C. The relationship between childhood maltreatment and non-suicidal self-injury in adolescents with depressive disorders. Psychiatry Res. (2024) 331:115638. doi: 10.1016/j.psychres.2023.115638, PMID: 38035534

[37] Markus HR Kitayama S. Culture and the self: Implications for cognition, emotion, and motivation. psychol Review. (2014) 98:224–53. doi: 10.1037/0033-295X.98.2.224

[38] Zhao SQ Zhang SC Wang H. Validity and reliability of the Emotion Awareness Questionnaire in middle school students. Chin Ment Health J. (2022) 36:597–602. doi: 10.3969/j.issn.1000-6729.2022.07.009

[39] Qiao C Lin J Huang J Zhou L Huang Y Shang Y. An exploration of the association between family functioning and nonsuicidal self-injury among Chinese adolescents with mood disorders. Eur J Psychiatry. (2024) 38:100226. doi: 10.1016/j.ejpsy.2023.100226

[40] Yan H Yue W. Risk factors, theoretical models, and biological mechanisms of nonsuicidal self-injury: a brief review. Interdiscip Nurs Res. (2023) 2:112–20. doi: 10.1097/nr9.0000000000000023, PMID: 37645376 PMC10461723

[41] Niedtfeld I Schulze L Kirsch P Herpertz SC Bohus M Schmahl C. Affect regulation and pain in borderline personality disorder: a possible link to the understanding of self-injury. Biol Psychiatry. (2010) 68:383–91. doi: 10.1016/j.biopsych.2010.04.015, PMID: 20537612

[42] Plener PL Bubalo N Fladung AK Ludolph AG Lulé D. Prone to excitement: adolescent females with Non-suicidal self-injury (NSSI) show altered cortical pattern to emotional and NSS-related material. Psychiatry Res. (2012) 203:146–52. doi: 10.1016/j.pscychresns.2011.12.012, PMID: 22901627

[43] Lin SC Pozzi E Kehoe CE Havighurst S Schwartz OS Yap MBH. Family and parenting factors are associated with emotion regulation neural function in early adolescent girls with elevated internalizing symptoms. Eur Child Adolesc Psychiatry. (2024) 33(12):4381–91. doi: 10.1007/s00787-024-02481-z, PMID: 38832959 PMC11618192

[44] Zhuo R Yu Y Shi X. Family resilience and adolescent mental health during COVID-19: A moderated mediation model. Int J Environ Res Public Health. (2022) 19:4801. doi: 10.3390/ijerph19084801, PMID: 35457666 PMC9028193

[45] Boyes ME Mah MA Hasking P. Associations between family functioning, emotion regulation, social support, and self-injury among emerging adult university students. J Child Fam Stud. (2023) 32:846–57. doi: 10.1007/s10826-022-02516-6

